# Dramatic regression of mass-forming gastric IgG4-related disease with inebilizumab: a case report

**DOI:** 10.1186/s12876-026-05139-z

**Published:** 2026-07-25

**Authors:** Rami Jadallah, Vikrant Singh, Nasir Notta, Anisha Dash, Abdul Wasay, Paul Eberts, Shaker Barham

**Affiliations:** 1 Department of Medicine, University of Tennessee Health Science Center, Knoxville, USA; 2 Department of Pathology, University of Tennessee Health Science Center, Knoxville, USA

**Keywords:** Gastric IgG4-related disease, Inebilizumab, CD19-directed therapy, B-cell depletion, Mass-forming gastric lesion, Hypereosinophilia, Endoscopic mucosal resection, Glucocorticoid-sparing therapy, Autoimmune pancreatitis, Storiform fibrosis, Rituximab, MITIGATE trial, Nonischemic cardiomyopathy, *Helicobacter pylori*

## Abstract

**Background:**

Gastric immunoglobulin G4-related disease (IgG4-RD) is exceedingly rare, with fewer than 50 cases reported worldwide. Its mass-forming presentation frequently mimics malignancy, leading to high rates of unnecessary surgical resection. Inebilizumab, a CD19-directed monoclonal antibody and the first FDA-approved therapy for IgG4-RD, has not previously been described in gastric IgG4-RD. We report the first case of mass-forming gastric IgG4-RD treated with inebilizumab, achieving clinical, endoscopic, and histologic remission without surgical intervention.

**Case presentation:**

A 52-year-old man with chronic hypereosinophilia, nonischemic cardiomyopathy, and multisystem inflammatory disease presented with symptomatic anemia and a 2 cm gastric antral mass identified on CT imaging. Endoscopy revealed an antral mass, highly suspicious for malignancy. Initial biopsies showed *Helicobacter pylori* associated chronic active gastritis without definitive malignancy. Repeat sampling after *H. pylori* eradication demonstrated dense transmural lymphoplasmacytic and eosinophilic infiltration with 225 IgG4⁺ plasma cells per high-power field, an IgG4/IgG ratio of 37.5%, and a markedly elevated serum IgG4 of 1,245 mg/dL, establishing the diagnosis of gastric IgG4-RD. Concurrent lung biopsy confirmed multisystem involvement. The patient was initiated on inebilizumab. Within six weeks, follow-up endoscopy demonstrated significant resolution of the gastric mass with histologic remission (IgG4/IgG ratio < 10%), and six-month follow-up revealed near-complete resolution of the gastric abnormalities.

**Conclusions:**

This case demonstrates that CD19-targeted B-cell depletion with inebilizumab can induce rapid, profound remission in gastric IgG4-RD, obviating the need for gastrectomy and chronic glucocorticoid therapy. These findings support early consideration of IgG4-RD in the differential diagnosis of mass-forming gastric lesions and highlight the transformative potential of mechanism-based therapy in this diagnostically challenging condition.

## Background

Immunoglobulin G4-related disease (IgG4-RD) is a fibroinflammatory multisystem disorder characterized by tumefactive lesions, IgG4-rich lymphoplasmacytic infiltrates, storiform fibrosis, and obliterative phlebitis [1, 2]. IgG4-RD is now understood as a unified disease process capable of affecting virtually any organ system through antigen-driven interactions between CD4 + T cells, B cells, and IgG4-expressing plasmablasts [3]. Although it can affect nearly any organ, the pancreas, biliary tree, salivary and lacrimal glands, retroperitoneum, kidneys, and lungs are commonly involved [1, 2, 4]. Despite its diverse clinical manifestations, characteristic histopathologic findings provide a consistent diagnostic framework [1, 5].

Gastrointestinal involvement is uncommon, and gastric IgG4-RD is particularly rare. A Japanese multicenter series identified only 12 gastric cases, while a systematic review documented just 42 reported cases worldwide through 2021 [6–8]. Gastric IgG4-RD often presents as a mass-forming lesion that mimics gastric adenocarcinoma, gastrointestinal stromal tumor (GIST), or lymphoma, frequently leading to unnecessary surgical resection [7–10]. Diagnosis can be challenging because superficial biopsies may miss the characteristic submucosal or transmural infiltrates, often necessitating deep tissue acquisition by endoscopic mucosal resection or fine-needle biopsy [11, 12]. Eosinophilia is a recognized feature of IgG4-RD, particularly in gastrointestinal disease, and an association with Helicobacter pylori has been reported, although a causal relationship remains unproven [11–16].

Historically, management has relied on glucocorticoids, but relapses and treatment-related toxicity have driven interest in B-cell-targeted therapies [17–19]. CD20-directed rituximab has shown efficacy in observational studies with no randomized trial data support for its use in IgG4-RD [20]. Inebilizumab, a CD19-directed monoclonal antibody, demonstrated efficacy in the MITIGATE trial and became the first FDA-approved therapy for IgG4-RD in 2025 [21]. We report the first case of mass-forming gastric IgG4-RD successfully treated with inebilizumab, achieving clinical, endoscopic, and histologic remission without surgery or chronic glucocorticoid exposure.

## Case presentation

A 52-year-old man with a medical history including chronic hypereosinophilia, nonischemic cardiomyopathy status post automated implantable cardioverter-defibrillator (AICD) placement, asthma-COPD overlap syndrome, current cigarette smoker with a 35-pack-year smoking history and iron deficiency anemia first developed systemic symptoms in 2021. His presenting complaints included chronic diarrhea, progressive peripheral neuropathy, exertional dyspnea, and profound fatigue. Laboratory evaluation revealed persistent microcytic anemia and marked peripheral eosinophilia.

Given the unexplained hypereosinophilia, he underwent extensive hematologic evaluation from 2021 to 2022, including bone marrow biopsy, serum protein electrophoresis with free light chains (SPEP/FLC), and comprehensive molecular testing for myeloproliferative neoplasms (JAK2, CALR, MPL mutations) and eosinophilia-associated genetic abnormalities (BCR-ABL1, PDGFRA, PDGFRB, FGFR1 rearrangements, and eosinophilia FISH panel). No clonal, malignant, or secondary hematologic cause of eosinophilia was identified. Over the subsequent years, the patient received multiple intermittent courses of prednisone from various providers, including hematology/oncology for the unexplained eosinophilia and pulmonology for episodic COPD exacerbations. Dosing typically ranged from 20 to 40 mg daily for periods of a few days to several weeks, with tapering and exact regimens that varied by provider and were not fully captured in the medical record. Each course produced consistent but temporary clinical and laboratory improvement, strongly suggesting a steroid-responsive inflammatory disorder. In April 2021, for evaluation of iron deficiency anemia, an esophagogastroduodenoscopy (EGD) and colonoscopy were performed. EGD revealed severe gastritis with mucosal inflammation and erosions, prompting initiation of proton pump inhibitor therapy. Colonoscopy revealed a 5 mm descending colon tubular adenoma removed via hot snare and left sided diverticulosis. In late 2021, the patient developed severe nonischemic cardiomyopathy with left ventricular ejection fraction (LVEF) of 15% and global hypokinesis on echocardiography. Coronary angiography demonstrated normal coronary arteries, excluding ischemic etiology. Given the temporal association with marked eosinophilia, eosinophilic myocarditis was suspected. Cardiac MRI performed during the cardiomyopathy workup showed moderately dilated left ventricle with normal thickness and severe global systolic dysfunction, EF 11.6%. There was no evidence of iron overload, but it demonstrated subendocardial edema in the mid and apical walls with diffuse linear subendocardial late gadolinium enhancement of the entire myocardium; These findings were most consistent with eosinophilic myocarditis by then. Further findings noted normal right ventricular size with moderate to severe systolic dysfunction with no significant valvular abnormalities. Endomyocardial biopsy performed shortly thereafter showed only myocyte hypertrophy without significant inflammation, fibrosis, or amyloid deposition, and no definitive eosinophilic infiltration was identified. The patient underwent AICD implantation in April 2022 and was initiated on guideline-directed medical therapy (GDMT) for heart failure.

Over the subsequent two years, serial computed tomography (CT) imaging revealed evolving systemic manifestations including pulmonary nodules, pleural effusions, and mural thrombi in the infrarenal aorta and iliac arteries, findings suggestive of a progressive systemic inflammatory and potentially vasculitic process. The constellation of hypereosinophilia, multiorgan involvement, vascular complications, and steroid responsiveness raised suspicion for a systemic immune-mediated disorder, though a unifying diagnosis remained elusive. Serologic evaluation for EGPA showed negative MPO-ANCA and PR3-ANCA, but total serum IgE was mildly elevated at 593. Unfortunately, the patient was subsequently lost to follow-up.

**Fig. 1:**
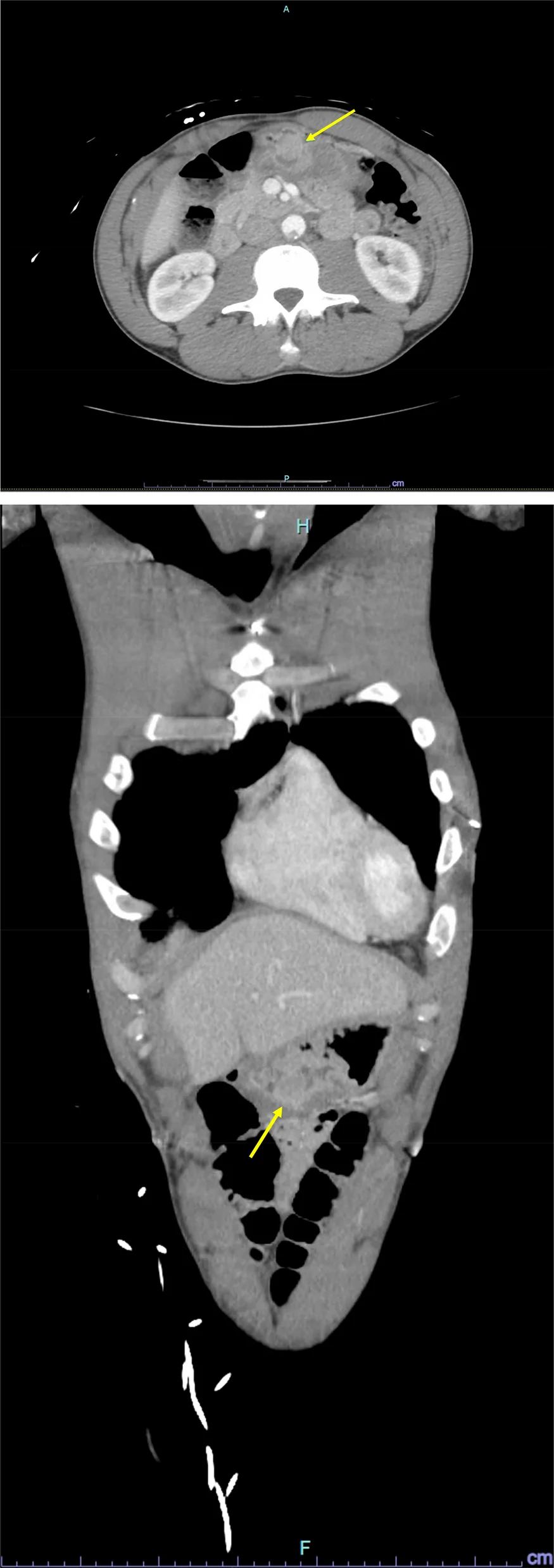
Axial and Coronal CT Thorax Abdomen Pelvis with IV contrast showing 2 cm Gastric mass highly concerning for Malignancy

**Fig. 2:**
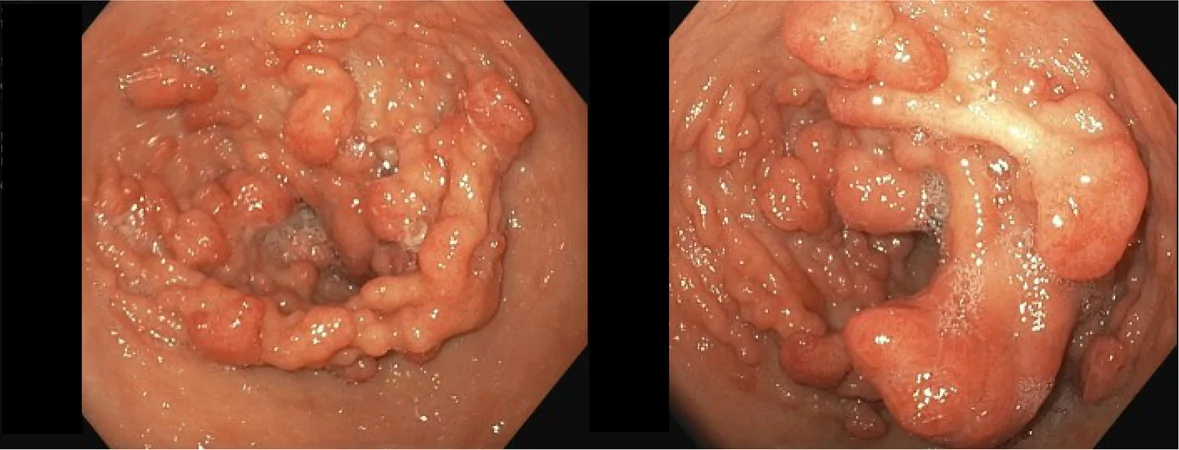
Endoscopic examination showing circumferential nodular mucosa with friable tissue and mass-like deformity in the gastric antrum

In late March 2025, the patient presented to the emergency department with a near-syncopal event and symptomatic anemia (hemoglobin 5.9 g/dL). CT imaging demonstrated a 2 cm mass-like lesion in the gastric antrum/pylorus, raising concern for malignancy (Fig. 1). EGD revealed a large, polypoid circumferential mass with oozing bleeding and stigmata of recent bleeding in the gastric antrum (Fig. 2). Biopsies demonstrated H. pylori-associated chronic active gastritis with reactive epithelial atypia and no definitive malignancy. At this presentation, total serum IgE had normalized to 401; IgE was not trended thereafter. Surgical oncology was consulted and recommended endoscopic ultrasound (EUS) for further characterization and staging prior to possible antrectomy.

**Fig. 3:**
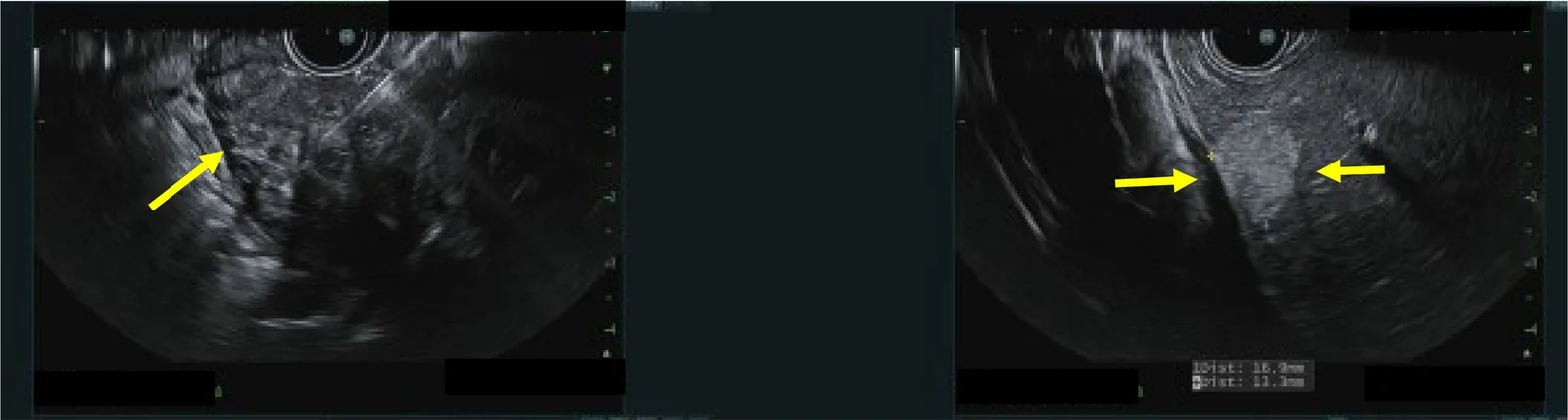
Endoscopic ultrasound image revealing antral mass with involvement of MP layer (left) and left liver lobe lesion (right)

EUS a few days later demonstrated a heterogeneous hypoechoic process involving the mucosa and submucosa with possible muscularis propria (MP) involvement, without definitive lymphadenopathy. There was a 16 mm hyperechoic lesion in the left lobe of the liver (Fig. 3). The EUS findings were suspicious for adenocarcinoma. To obtain deeper tissue and maximize yield for diagnosis, EUS guided fine needle biopsy in addition to endoscopic mucosal resection (EMR) of large parts of the mass. The liver lesion was biopsied via fine needle aspiration and cytology revealed blood and patchy mixed inflammation. Upon review of previous abdominal MRI from 2021, the left lobe lesion was determined to be a hemangioma and measured 16 mm at that time as well. Histopathologic examination of resected specimens demonstrated an inflammatory fibroid polyp with a dense mixed inflammatory infiltrate, without evidence of malignancy or gastrointestinal stromal tumor (GIST). IgG4-RD was not initially considered. The patient was subsequently initiated on *H. pylori* triple therapy (amoxicillin, clarithromycin and omeprazole) for 14 days. Concurrently, for management of COPD exacerbation during the same admission he was treated with 5 days of prednisone 40 mg daily.

**Fig. 4:**
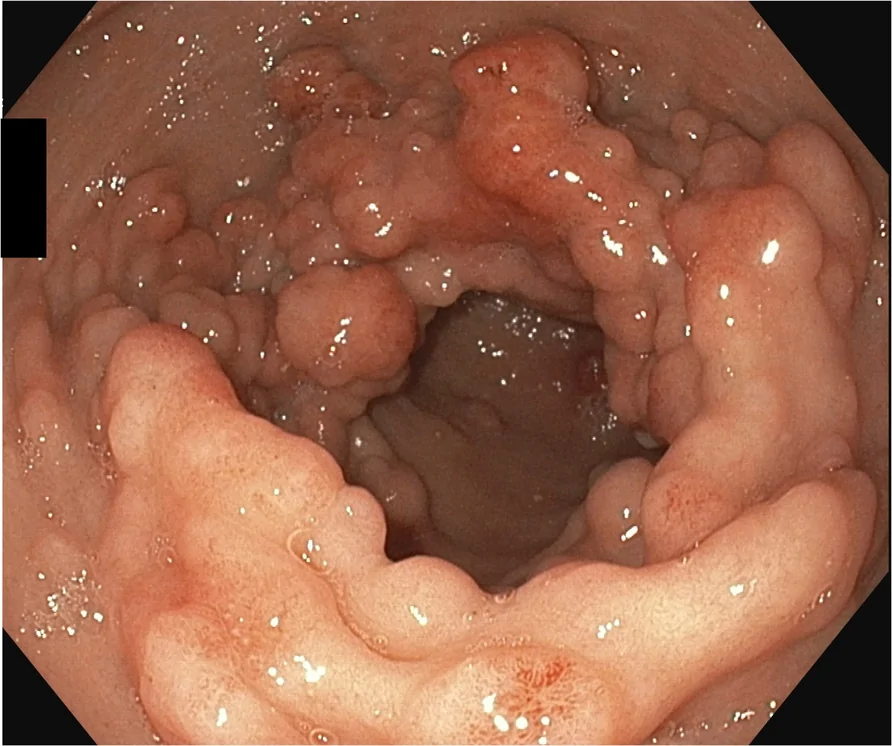
Endoscopic view showing persistent mass-like inflammatory changes in the gastric antrum

Repeat EGD in June 2025 demonstrated persistent mass-like inflammatory changes in the gastric antrum with nodular, edematous mucosa. Extensive tissue sampling was again obtained using EMR technique; however, complete resection was not feasible due to the lesion’s size (Fig. 4). Obtained specimens again ruled out malignancy and were negative for H. pylori gastritis confirming successful eradication after triple therapy.

**Fig. 5:**
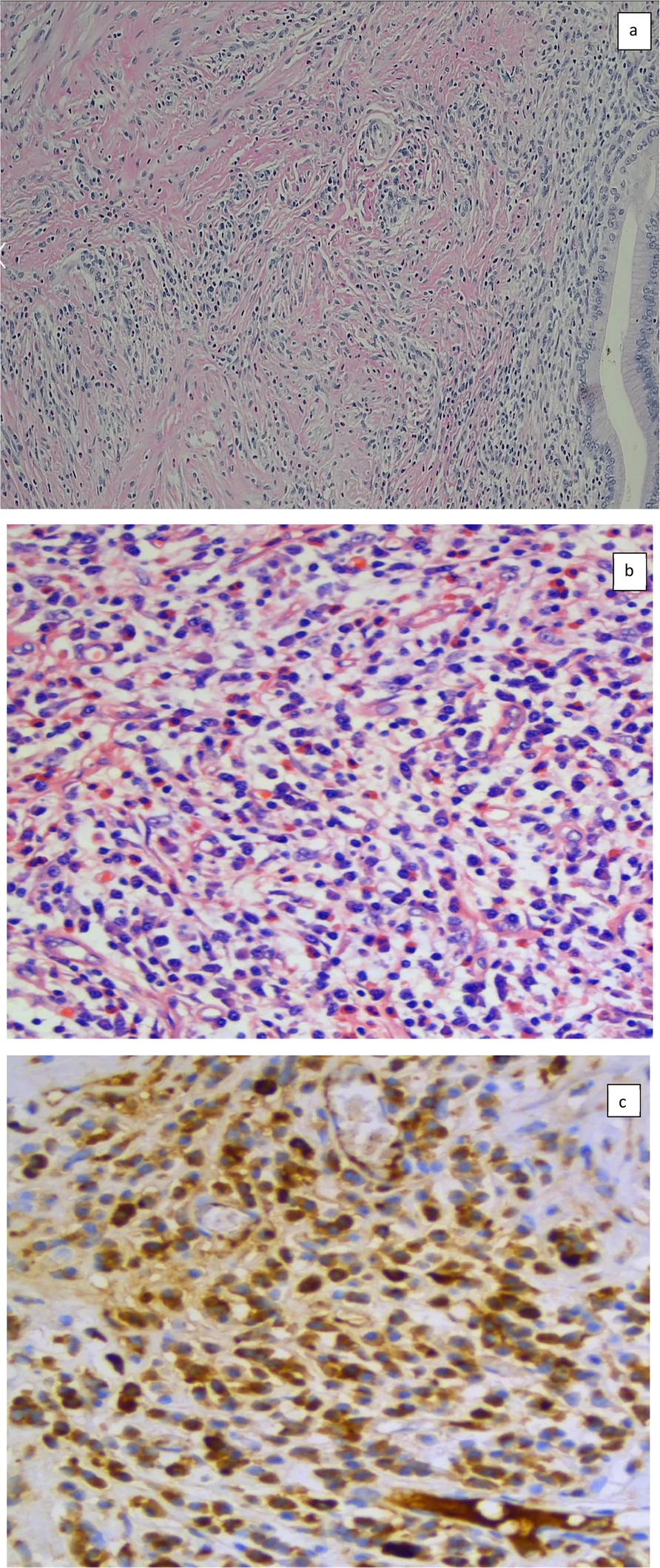
**a** H&E 100x: Gastric sections demonstrate classic storiform fibrosis with a prominent lymphoplasmacytic inflammatory cell infiltrate. **b** H&E 200x- Numerous plasma cells with background eosinophils. **c** IgG4 IHC stain 200x- Numerous IgG4-positive plasma cells identified by immunohistochemistry for IgG4

At that time, possibility of IgG4-RD was raised with the pathology department as part of the differential diagnosis. Histopathologic evaluation later revealed dense transmural and submucosal eosinophilic and plasmacytic infiltration with focal storiform fibrosis, consistent with the disease process. Immunohistochemical staining showed 600 IgG⁺ plasma cells per high-power field (HPF) and 225 IgG4⁺ plasma cells per HPF, corresponding to an IgG4/IgG ratio of 37.5% (Fig. 5). Serum total IgG was 3,492 mg/dL and serum IgG4 was markedly elevated at 1,245 mg/dL, Supporting the diagnosis. Given that not all classic histologic criteria were fully met, the case was independently reviewed by multiple pathologists within the department, who concurred with the diagnosis of IgG4-RD based on the totality of the histopathologic, serologic, and clinical findings. The complete longitudinal laboratory data across the disease course, including hemoglobin, absolute eosinophil count, total IgG, serum IgG4, inflammatory markers, and serologic testing, are summarized in (Table 1).

**Table 1:** Comprehensive Laboratory Timeline Across the Disease Course

Date	Disease phase/clinical event	Hgb (g/dL)	Abs eos (× 10^3^/μL)	Total IgG (mg/dL)	Serum IgG4 (mg/dL)	ESR (mm/hr)	Other testing/serology (see footnote for reference ranges)
Mar 2021	Earliest available CBC; intermittent eosinophilia recognized	10.9 (L)	2.4 (H)	-	-	-	-
Jul-Sep 2021	Hypereosinophilia workup; symptomatic anemia	6.6–11.2 (L)	1.5–5.9 (H)	-	-	-	Range across multiple draws; eosinophil peak 5.9 on 8 Sep 2021
Nov 2021	Post-prednisone course	11.8 (L)	0.0	-	-	-	Eosinophils transiently suppressed on steroids
Dec 2021	Cardiomyopathy/autoimmune workup	13.1	0.4	-	-	30 (H)	Anti-DsDNA < 1, anti-Sm < 0.2, anti-SSA < 0.2, anti-SSB < 0.2, anti-RNP < 0.2 (all negative)
May 2022	Inter-current evaluation	16.0	3.7 (H)	1,483; 1,440 (H)	-	-	SPEP: gamma 1.5; M-spike 0.7 H; total IgE 593 kU/L (H)
Feb 2023	Allergy/atopy workup	-	-	-	-	-	IgE allergen panel—maple leaf sycamore, hickory pecan, white oak, Aspergillus fumigatus all < 0.10 (negative)
Mar 2025	ED presentation—near-syncope, symptomatic anemia	5.9 (critical)	0.6 (H)	-	-	-	Triggered hospitalization and EGD
Mar 2025	Initial admission workup	7.4 (L)	0.8 (H)	2,358 (H)	-	> 120 (H)	ANCA panel—MPO-ANCA < 0.2, PR3-ANCA < 0.2, C-ANCA < 1:20, P-ANCA < 1:20 (all negative); Complement—C3 122, C4 21.4, CH50 54 (normal); SPEP—gamma 2.2 H; total IgE 401 kU/L (normal)
Jun 2025	Diagnosis of IgG4-RD on EMR specimen	9.4 (L)	2.2 (H)	3,492; 3,481 (H)	1,245 (H)	-	Pathology—225 IgG4⁺ plasma cells/HPF, IgG4/IgG ratio 37.5%; ENA panel repeated—all negative
Jul 2025	Pre-inebilizumab (peak eosinophilia)	10.1 (L)	12.9 (H)	-	-	-	Highest peripheral eosinophil count of the disease course
Aug 2025	Inebilizumab dose 1 (300 mg IV)	8.7 (L)	0.3	-	-	-	Concurrent prednisone taper for COPD exacerbation
Sep 2025	About 6 weeks post-induction (after dose 2)	14.8	1.4 (H, mild)	1,323	-	-	Hemoglobin normalized; IgG markedly reduced
Oct 2025	About 12 weeks post-induction	15.0	1.3 (H, mild)	1,288	-	-	∼63% reduction in total IgG from June 2025 peak
Feb 2026	Post-dose 3 (week 26 maintenance)	14.7	-	-	-	-	Sustained clinical, endoscopic remission

Reference ranges (institutional, Cerner EMR). Hemoglobin 13.5–17.5 g/dL (adult male); absolute eosinophil count 0.0–0.5 × 10^3^/μL; total serum IgG 700–1,600 mg/dL; serum IgG4 4–86 mg/dL (≥ 135 mg/dL supports IgG4-RD); ESR 0–20 mm/hr; C3 90–180 mg/dL; C4 10–40 mg/dL; CH50 30–75 U/mL; total serum IgE 6–495 kU/L. ANCA, ENA, and specific IgE values are reported as units used by the testing laboratory (EIA < 0.2 negative; immunofluorescence titer < 1:20 negative; specific IgE < 0.10 kU/L negative)

Abbreviations & legend. CBC, complete blood count; Hgb, hemoglobin; Eos, absolute eosinophil count; IgG, immunoglobulin G; IgG4, immunoglobulin G subclass 4; ESR, erythrocyte sedimentation rate; ANCA, antineutrophil cytoplasmic antibody; MPO, myeloperoxidase; PR3, proteinase 3; ENA, extractable nuclear antigen; SPEP, serum protein electrophoresis; SSA, anti-Sjögren’s syndrome A; SSB, anti-Sjögren’s syndrome B; EMR, endoscopic mucosal resection; EGD, esophagogastroduodenoscopy; HPF, high-power field; COPD, chronic obstructive pulmonary disease. H = above reference range; L = below reference range;—= not measured at this timepoint

**Fig. 6:**
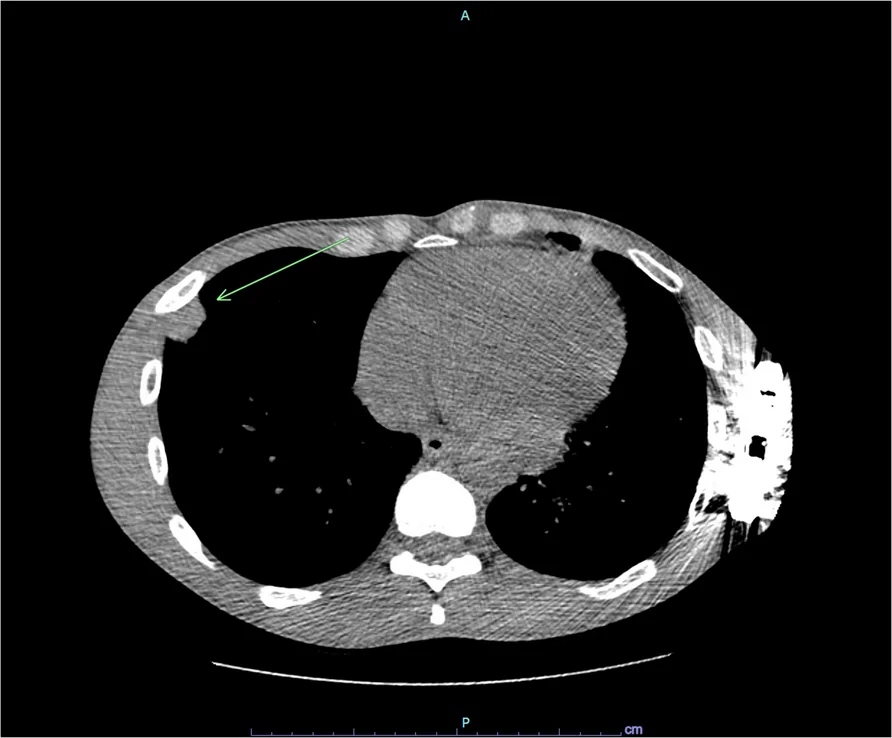
Axial CT Image showing subpleural nodule in the right middle lobe measuring up to approximately 1.8 cm

A lung biopsy of a right middle lobe nodule performed in June 2025 demonstrated scattered polytypic plasmacytic infiltrates (Fig. 6). Immunohistochemical staining revealed an IgG4/IgG ratio of 0.30, exceeding the established diagnostic cutoff of 0.147. Additionally, approximately 28 IgG4-positive plasma cells per high-power field were identified when averaged across the three most densely involved fields, exceeding the reported diagnostic threshold of 20 IgG4-positive plasma cells per high-power field. These findings supported pulmonary involvement by IgG4-RD and, together with the gastric pathology, confirmed multisystem disease involving the stomach and lungs, with possible vascular involvement given the history of aortic mural thrombi.

Given the systemic nature of his disease, multiorgan involvement, and a relapsing course despite multiple corticosteroid trials, as well as the availability of a newly FDA-approved targeted therapy, the patient was initiated on inebilizumab 300 mg intravenously on day 1 and day 15 (induction), with a planned third dose at week 26, in accordance with the MITIGATE trial protocol and the FDA-approved dosing regimen. Within 3 weeks of starting inebilizumab, the patient reported significant improvement in fatigue, dyspnea and gastrointestinal symptoms, including resolution of abdominal discomfort and normalization of bowel habits.

**Fig. 7:**
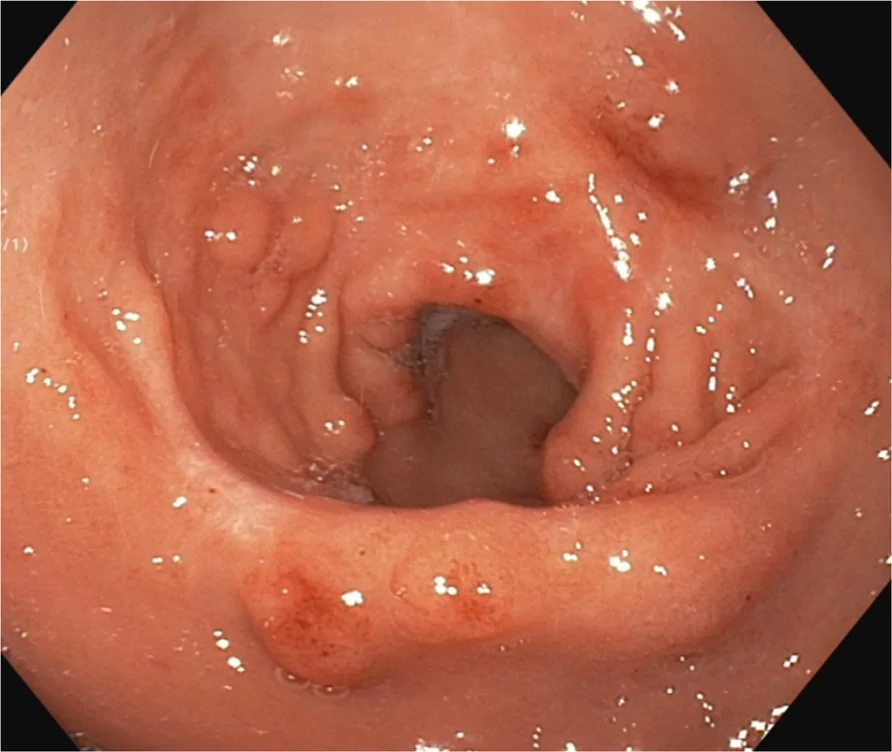
Endoscopic view in late 2025 showing regression of the gastric mass and nodularity

**Fig. 8:**
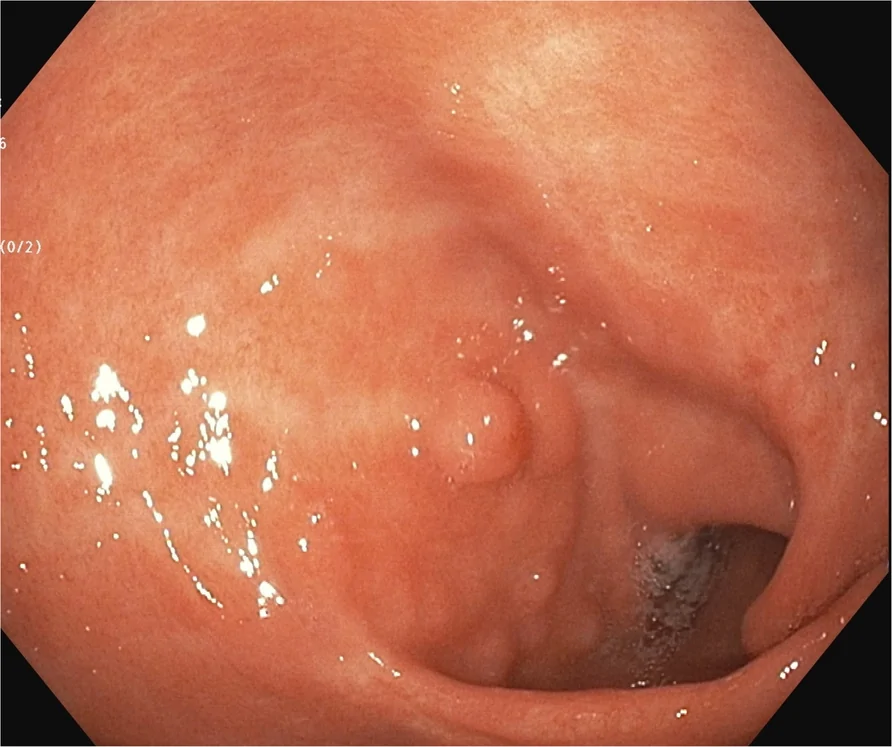
Endoscopic view of the gastric antrum in March 2026 showing near resolution of the mass

Follow-up EGD performed in September 2025—approximately 6 weeks after starting inebilizumab infusion—demonstrated dramatic regression of the gastric mass and nodularity, with major improvement of the prominent antral lesion. Residual mucosal erythema and mild irregularity were present, but the mass effect had mostly resolved (Fig. 7). Biopsies from the previously involved area revealed chronic gastritis with only minimal residual eosinophilic infiltrate, rare plasma cells on both H&E and immunohistochemical staining, and an IgG4/IgG ratio < 10%, consistent with histologic remission. Interval CT imaging demonstrated resolution of bilateral pleural effusions and complete resolution of the infrarenal aortic and iliac artery mural thrombi. Cardiac function improved significantly, with repeat echocardiography showing LVEF improvement from 15 to 35% on optimized heart failure medical therapy. The patient continued to have subjective improvement in his symptoms over the course of the next couple of months in addition to significant improvement in his anemia. Around early February 2026, he received the third dose of inebilizumab, followed by repeat EGD in March 2026 which showed almost complete resolution of his gastric mass (Fig. 8).

## Discussion

To our knowledge, this case represents the first reported use of inebilizumab for gastric IgG4-RD and illustrates a potential paradigm shift in management, achieving clinical, endoscopic, and histologic remission without surgical intervention or chronic glucocorticoid dependence. The patient’s dramatic response to CD19 targeted B-cell depletion underscores the central pathogenic role of B cells in IgG4-RD and further validates the therapeutic rationale for targeting earlier stages of B-cell development [21]. The clinical course at presentation reflects the diagnostic challenges that frequently precede definitive therapy. The initial endoscopic findings were concerning for malignancy, prompting surgical consultation and consideration of gastrectomy. This scenario mirrors the broader experience reported in the literature, where approximately half of patients with gastric IgG4-RD undergo surgical resection, with the diagnosis often established only after postoperative pathologic evaluation [8]. Such diagnostic uncertainty is compounded by the limitations of superficial endoscopic biopsies, which frequently fail to capture the characteristic submucosal or transmural infiltrates. As demonstrated in this case, deep tissue acquisition through endoscopic mucosal resection was essential to reveal the pathognomonic lymphoplasmacytic infiltration [11, 12]. Notably, the circumferential nodular, mass-like deformity observed in our patient differs from the endoscopic patterns most frequently reported in gastric IgG4-RD, which are predominantly ulcerative or submucosal tumor-like in appearance; mass-forming and nodular presentations such as this are less commonly described and further contribute to the diagnostic difficulty [8, 10].

Beyond its mass-forming presentation, this case was further complicated by longstanding hypereosinophilia, which initially obscured the diagnostic picture. Despite extensive hematologic evaluation that excluded a clonal or malignant process, the persistence of peripheral eosinophilia ultimately proved to be a critical diagnostic clue. The presence of both peripheral and tissue eosinophilia aligns with current literature recognizing eosinophilia as a frequent yet underappreciated feature of IgG4-RD, particularly in gastrointestinal involvement [13, 14]. Importantly, patients with eosinophilia have been shown to exhibit more extensive organ involvement, higher serum IgG4 levels, and increased relapse rates [22, 23]. Consistent with these findings, this patient demonstrated multi-organ diseases involving the stomach, lungs, and potentially the cardiovascular system. The constellation of peripheral and tissue eosinophilia, pulmonary nodules, vascular mural thrombi, and elevated serum IgG4 raises a substantive differential. Eosinophilic granulomatosis with polyangiitis (EGPA) merits particular consideration, as serum IgG4 can be elevated in approximately 30% of patients with EGPA [24, 25]. Gastrointestinal involvement (including eosinophilic gastroenteritis) is also well described, and the patient’s history of asthma-COPD overlap, peripheral neuropathy, and pulmonary involvement overlaps with the EGPA phenotype [26]. ANCA serologies at admission were negative (MPO-ANCA < 0.2, PR3-ANCA < 0.2, C-ANCA < 1:20, P-ANCA < 1:20). Nonetheless, ANCA negativity does not exclude EGPA, particularly given that cardiac involvement is more commonly seen in ANCA-negative disease [27, 28]. Total serum IgE was only mildly elevated and later normalized. Additionally, no granulomatous or necrotizing small-vessel vasculitis was identified in the available histopathologic specimens, though vasculitis was not specifically evaluated as a primary diagnostic target. Sjögren disease was considered unlikely given the absence of sicca symptoms and the lack of imaging evidence for lacrimal or salivary gland involvement; an extended autoimmune serology panel was negative, including anti-SSA < 0.2, anti-SSB < 0.2, anti-Sm < 0.2, anti-RNP < 0.2, anti-dsDNA < 1, and normal complement (C3 122 mg/dL, C4 21.4 mg/dL). Multicentric Castleman disease was considered unlikely based on the absence of generalized lymphadenopathy on cross-sectional imaging and the histopathologic appearance of the biopsy specimens, which did not show features suggestive of Castleman disease [29]. Malignant lymphoma was considered unlikely based on the histopathologic appearance of a mixed lymphoplasmacytic and eosinophilic infiltrate without overt cytologic atypia or features suggestive of clonal lymphoid expansion. We acknowledge the legitimate phenotypic overlap with EGPA-spectrum disease in this patient and present the case while recognizing that diagnostic ambiguity; on balance, the gastric histopathology met established criteria for IgG4-RD. Specifically, when IgG4-RD was suspected during the diagnostic workup, the patient was prospectively assessed against the 2019 ACR/EULAR classification criteria, he satisfied the entry criterion of involvement of at least one of 11 possible organs in a manner consistent with IgG4-RD, did not meet any of the 32 exclusion criteria, and accumulated a score of 38 points, successfully satisfying the 2019 ACR/EULAR classification criteria for IgG4-Related Disease (cutoff ≥ 20 points) with an established sensitivity of 82.0%−85.5% and a specificity of 97.8%−99.2% for IgG4-RD [30]. The systemic response to CD19-directed B-cell depletion is most consistent with that diagnosis.

The potential cardiovascular involvement further emphasizes the systemic nature of this disease process. The patient’s severe nonischemic cardiomyopathy, with an initial left ventricular ejection fraction (LVEF) of 15%, demonstrated temporal association with marked eosinophilia and subsequently improved following immunosuppressive therapy. Although endomyocardial biopsy did not reveal definitive eosinophilic infiltration or the classic histopathologic features of IgG4-RD, cardiovascular manifestations of IgG4-RD are increasingly recognized and include aortitis, pericarditis, myocardial disease, and valvulopathy [31, 32]. Cardiac MRI findings were highly suggestive of eosinophilic cardiomyopathy. Despite a nondiagnostic endomyocardial biopsy, eosinophilic cardiac involvement remained a strong consideration, as the patchy nature of the disease is associated with a reported biopsy sensitivity of only approximately 54% [33]. The presence of mural thrombi within the infrarenal aorta and iliac arteries, which resolved on follow-up imaging after inebilizumab therapy, further supports systemic vascular involvement. Notably, improvement in cardiac function from an LVEF of 15% to 35% following targeted B-cell depletion suggests that the underlying inflammatory process, whether directly IgG4-RD mediated or eosinophil-driven, was responsive to this therapeutic strategy.

Establishing the diagnosis required careful integration of clinical, serologic, and histopathologic findings. We acknowledge that several of the supportive findings are, in isolation, non-specific: the tissue IgG4/IgG ratio of 37.5% falls just below the conventional 40% threshold, and a markedly elevated serum IgG4 may be seen in other eosinophilic conditions, including approximately one-third of EGPA cases [24, 25]. The demonstration of storiform fibrosis, a dense lymphoplasmacytic infiltrate, 600 IgG + plasma cells per high-power field, 225 IgG4 + plasma cells per high-power field, and an IgG4/IgG ratio of 37.5% must be interpreted within the context of gastric tissue, where diagnostic thresholds may vary, and as a constellation rather than as isolated criteria [2, 12]. The concurrent presence of H. pylori associated gastritis adds another layer of complexity. While this association with IgG4-RD has been described in multiple case series and has prompted consideration of a pathogenic role through molecular mimicry, prospective cohort data have not supported this mechanism [11, 15].

With the diagnosis secured and systemic involvement recognized, the patient’s response to targeted therapy further clarifies the therapeutic implications of this case. Symptomatic improvement occurred within 2–3 weeks of initiating inebilizumab, followed by marked endoscopic and histologic regression at week six. Within the first few months of therapy, there was near-complete resolution of the gastric mass, reduction of the IgG4/IgG ratio to < 10%, resolution of pleural effusions, and disappearance of vascular thrombi. This clinical trajectory parallels pharmacodynamic data from the MITIGATE trial, which demonstrated rapid B-cell depletion within 2–4 weeks and progressive reductions in serum IgG4 levels reaching approximately 50–70% by week 12 [21, 34]. The sustained improvement in cardiac function further reinforces the systemic impact of B-cell depletion.

The attribution of clinical improvement specifically to inebilizumab merits careful consideration because the patient received two concurrent interventions during the relevant interval: a short course of prednisone for COPD exacerbation, and *H. pylori* eradication therapy. Both have the potential to confound the assessment of response, since corticosteroids can independently induce remission in IgG4-RD and EGPA-spectrum disease, and *H. pylori* eradication can improve gastric mucosal abnormalities [35, 36]. Several observations argue against either being the primary driver of the response in this case. The patient had received multiple prior intermittent prednisone courses of comparable dose (20–40 mg daily for days to several weeks) over the preceding years, notably below conventional induction dosing for IgG4-RD of 0.6–1 mg/kg of prednisone equivalent for 2–4 weeks followed by gradual taper [35], with consistent but only temporary clinical and laboratory improvement and prompt recurrence on taper; an additional limited steroid course during the hospital admission in March is unlikely to account for the durable, near-complete endoscopic resolution and the histologic remission with reduction of the IgG4/IgG ratio to < 10%. With regard to *H. pylori*, eradication therapy was administered and repeat biopsies in June 2025 confirmed eradication; nevertheless, the mass-forming antral lesion persisted at that interval, indicating that H. pylori clearance alone did not account for resolution. Sustained resolution emerged only after inebilizumab induction, in parallel with deep B-cell depletion, normalization of hemoglobin (from a nadir of 5.9 g/dL to 14.7–15.0 g/dL), improvement of peripheral eosinophilia, and an approximately 63% reduction in total serum IgG (3,492 mg/dL pre-induction to 1,288 mg/dL by week 12). While we cannot exclude any contribution from concurrent therapies, the magnitude, durability, and multi-organ extent of the response are most consistent with a primary effect of CD19-directed B-cell depletion.

Mechanistically, the success of inebilizumab in this case reflects its unique targeting strategy. As a humanized, afucosylated IgG1 kappa monoclonal antibody directed against CD19, a marker expressed earlier in B-cell development than CD20 and persisting on plasmablasts and some plasma cells, inebilizumab offers theoretical advantages over rituximab, which targets CD20 and may not eliminate plasmablasts implicated in persistent disease activity. The MITIGATE trial, which led to FDA approval of inebilizumab in April 2025 as the first therapy for IgG4-RD, demonstrated an 87% reduction in flare risk compared with placebo. More participants receiving inebilizumab achieved flare-free, glucocorticoid-free complete remission at one year, with rapid, deep, and sustained peripheral B-cell depletion accompanied by progressive reductions in serum IgG4 levels, thereby mechanistically linking B-cell elimination to disease control [21, 37].

Several limitations of this case warrant transparent acknowledgment. First, the cardiovascular findings—particularly the LVEF improvement from 15 to 35% and the resolution of infrarenal aortic and iliac arterial mural thrombi—were not directly proven to be IgG4-RD mediated. The vascular abnormalities were not biopsied, and the contribution of optimized heart failure therapy and concurrent inflammatory suppression cannot be disentangled. The endoscopic improvement is, however, temporally consistent with the systemic response to inebilizumab. Second, serum IgG4 was quantified only once, at the time of diagnosis, and was not repeated after treatment; the serologic response was therefore tracked indirectly by total serum IgG rather than by serial IgG4 measurements, and a post-treatment serum IgG4 level would have more directly documented the serologic response. Similarly, total serum IgE was measured at only two timepoints and was not serially trended, limiting its utility as a longitudinal marker of disease activity. Third, although the lung biopsy demonstrated an elevated tissue IgG4/IgG ratio supporting pulmonary involvement, the histologic findings were relatively non-specific, representative lung histology slides were not available for illustration, and the pulmonary involvement therefore rests on the quantitative immunostaining and the imaging findings. Finally, the post-treatment IgG and IgG4 immunostained photomicrographs were of limited interpretive quality and have been omitted after Thorough discussion with the pathology department; because the IgG4 staining had been performed at an outside facility, the original slides could not be obtained for re-imaging or enhancement, and the post-treatment IgG4/IgG ratio of less than 10% is therefore reported on the basis of the formal pathology report rather than a reproducible image.

Taken together, this case highlights several important clinical lessons. Gastric IgG4-RD, although rare, can closely mimic malignancy and should be considered in the differential diagnosis of mass-forming gastric lesions, particularly in patients with eosinophilia, elevated serum IgG4 levels, or evidence of multisystem disease. Early recognition and deep tissue sampling may facilitate accurate diagnosis and prevent unnecessary surgical intervention. Furthermore, this case demonstrates the potential of CD19-targeted B-cell depletion with inebilizumab to induce rapid, durable, glucocorticoid-sparing remission in patients with multisystem IgG4-RD, supporting its role as a promising targeted therapeutic strategy [17, 37].

## Conclusion

To our knowledge, this case represents the first published report of inebilizumab use in gastric IgG4-related disease. The profound response observed across clinical, endoscopic, histologic, and systemic manifestations supports the emerging role of CD19-directed B-cell depletion as a rational therapeutic strategy for IgG4-RD. Beyond its therapeutic implications, this case highlights the diagnostic challenges posed by gastric IgG4-RD and reinforces the importance of maintaining clinical suspicion for this entity in patients presenting with mass-forming gastric lesions. Early measurement of serum IgG4 levels, together with deep tissue acquisition and multidisciplinary evaluation, may facilitate a more timely diagnosis, avoid unnecessary surgical resection, and expedite initiation of targeted therapy.

## Data Availability

All data generated or analyzed during this case report are included in this published article. Further inquiries regarding the case can be directed to the corresponding author upon reasonable request, in accordance with patient confidentiality and institutional policies.

## Abbreviations

ACR: American College of Rheumatology
AICD: Automated implantable cardioverter-defibrillator
ANCA: Antineutrophil cytoplasmic antibody
CD: Cluster of differentiation
COPD: Chronic obstructive pulmonary disease
CT: Computed tomography
EGD: Esophagogastroduodenoscopy
EGPA: Eosinophilic granulomatosis with polyangiitis
EMR: Endoscopic mucosal resection
ENA: Extractable nuclear antigen
EUS: Endoscopic ultrasound
EUS-FNA: Endoscopic ultrasound-guided fine-needle aspiration
EUS-FNB: Endoscopic ultrasound-guided fine-needle biopsy
EULAR: European Alliance of Associations for Rheumatology
FDA: U.S. Food and Drug Administration
FISH: Fluorescence in situ hybridization
GIST: Gastrointestinal stromal tumor
H&E: Hematoxylin and eosin
HPF: High-power field
IgG: Immunoglobulin G
IgG4-RD: Immunoglobulin G4-related disease
IHC: Immunohistochemistry
IRB: Institutional Review Board
IV: Intravenous
LVEF: Left ventricular ejection fraction
MITIGATE: Multinational Investigation in IgG4-RD Therapy with Inebilizumab Gauging Adverse Events and Efficacy (trial)
MPO: Myeloperoxidase
MRI: Magnetic Resonance Imaging
PR3: Proteinase 3
RNP: Ribonucleoprotein
SPEP/FLC: Serum protein electrophoresis with free light chains
SSA: Anti-Sjögren’s syndrome A
SSB: Anti-Sjögren’s syndrome B
